# Uncertainties in Screening and Prevention of Group B Streptococcus Disease

**DOI:** 10.1093/cid/ciy1069

**Published:** 2018-12-17

**Authors:** Kirsty Le Doare, Paul T Heath, Jane Plumb, Natalie A Owen, Peter Brocklehurst, Lucy C Chappell

**Affiliations:** 1 Paediatric Infectious Disease Research Group & Vaccine Institute, St George’s University of London, London, United Kingdom; 2 Group B Strep Support, London, United Kingdom; 3 Science, Research and Evidence, Department of Health and Social Care, London, United Kingdom; 4 Birmingham Clinical Trials Unit, University of Birmingham, London, United Kingdom; 5 Department of Obstetrics, King’s College London, London, United Kingdom

**Keywords:** group B streptococcus, screening, maternal, neonatal, vaccine

## Abstract

In autumn 2016, the UK Department of Health (now Department of Health and Social Care) convened 2 meetings to discuss how to address research evidence gaps in order to minimize the impact of infant group B streptococcus (GBS) disease in the United Kingdom. At that meeting, a number of research priorities were highlighted, including improving the screening for GBS colonization in pregnant women, offering intrapartum antibiotic prophylaxis and point-of-care testing, and understanding the effect of widespread intrapartum antibiotic use on long-term infant health. Further discussions involved investigating the feasibility of a large prospective study of pregnant women and their infants in order to understand the role of antibodies in the protection against GBS disease in infancy following maternal exposure to GBS colonization. Here, we summarize the research uncertainties identified at that meeting.

## Teaser

We present research gaps related to group B streptococcus screening strategy, disease prognostic factors, serocorrelates of protection, and women’s attitudes regarding screening and prevention strategies. We provide clearly identified research priorities to stimulate researchers and funders to address these gaps.

Group B streptococcus (GBS) is a bacterium carried in the urogenital and gastrointestinal or urinary tract in the general population. However, approximately 20% of pregnant women carry GBS at any one time, and there is a risk to the infant associated with passage of the bacterium from mother to infant at birth [1]. GBS can cause septicemia, pneumonia, meningitis, and death in up to 2% of infants born to colonized women in the absence of intrapartum antibiotic prophylaxis (IAP) [2]. GBS disease primarily occurs in babies in 2 forms: early onset (EOGBS), occurring between birth and day 6 of life, and late onset (LOGBS), occurring from day 7 to day 90 of life, with the disease practically disappearing thereafter. The risk factors associated with LOGBS are poorly understood. Survivors of GBS disease have a higher risk of long-term neurodevelopmental impairment (particularly following meningitis) that can severely impact quality of life [3]. In the United Kingdom, a recent national surveillance study showed that the incidence of culture-confirmed EOGBS disease appears to be rising, from 0.48/1000 (95% confidence interval [CI], 0.43–0.53) live births in 2000 to 0.57/1000 (95% CI, 0.52–0.62) in 2014, despite a clinical risk factor–based IAP policy introduced in 2003 [4]. Over the same period of time, LOGBS incidence also appears to have risen from 0.24/1000 (95% CI, 0.21–0.28) to 0.37/1000 (95% CI, 0.33–0.41) live births. GBS is now the leading cause of severe bacterial infection in neonates [5] and of bacterial meningitis [4] in UK infants. Several unanswered questions exist concerning how to prevent all forms of GBS infection.

Policies for GBS screening vary among countries, with some offering universal screening (eg, through microbiological testing) to all pregnant women and others undertaking this selectively [6]. In the United Kingdom, the Royal College of Obstetrics and Gynaecology (RCOG) [7] and the National Institute for Health and Care Excellence [8] recommendations have been to offer IAP to women identified as having the following risk factors for GBS: previous baby with invasive GBS infection, GBS bacteriuria in the current pregnancy, vaginal or rectal swab positive for GBS in the current pregnancy, or maternal intrapartum pyrexia above 38°C. However, the recent national surveillance study showed that only 35% of a cohort of 429 UK and Irish cases with EOGBS disease had 1 or more of these risk factors compared to 65% in the 2000–2001 study [9]. Since this surveillance was undertaken, the RCOG guidelines have been updated (in 2017) [7]. These revised guidelines now include preterm labor as an additional risk factor for the offer of IAP and a recommendation for women who have previously had a positive GBS result to either be treated as a carrier in the current pregnancy or to offer repeat testing and IAP if appropriate. This could potentially add up to 12% more women to the proportion who might be offered IAP [7]. Nevertheless, a risk factor–based approach provides only limited protection against invasive EOGBS disease in the infant.

In around half of high-income countries, women who are not already identified as at increased risk of their baby developing EOGBS are offered a test for GBS colonization at 35–37 weeks of gestation (“universal screening”), with subsequent IAP for those whose swab is positive for GBS (around 18%–20%) [6]. Both the risk-based and universal screening approaches entail giving IAP to a substantial proportion of pregnant women, the great majority of whom would not have had an affected baby even without the IAP.

Further uncertainties exist in how best to detect those infants at risk of both EOGBS and LOGBS infection. Current test-based screening is developed to identify maternal GBS colonization rather than neonatal or infant invasive disease. The risk factors used to identify women who should be offered IAP are generic clinical risk factors and are not sensitive or specific for EOGBS.

A recent systematic review concluded that the evidence base that addresses the potential impact of widespread use of IAP for the mother (anaphylaxis, antimicrobial resistance, medicalization of labor) and baby (short- and long-term effects on the gut microbiome, longer stay in hospital, antimicrobial resistance) was limited and that additional large, high-quality, and longitudinal observational studies across countries would improve our understanding in this area [10]. Given that in the United Kingdom approximately 20% of women are colonized yet only 1 in 100 infants born to women carrying GBS develop invasive GBS disease [4], there is a need to understand other factors that contribute to natural protection from infection, such as antibody concentrations. Such data would also facilitate vaccine development and licensure.

A recently published collection of articles highlights the potential global burden and the scale of the problem [2]. Independently (though with overlap with some of the experts), the UK Department of Health (now Department of Health and Social Care) convened a working group to examine some of the research uncertainties that could be tackled in the United Kingdom with appropriately commissioned funding. Here, we present a consensus view on current research gaps in evidence on screening, diagnostic tests, and vaccination, with the aim of stimulating research in this area.

## SCREENING APPROACHES FOR GBS

Two main approaches to screening pregnant women are widely practiced in high- and middle-income settings: universal screening or risk factor based–screening. The Centers for Disease Control and Prevention, American College of Obstetrics and Gynecologists, American Academy of Pediatrics, American Society for Microbiology, and American College of Nurse-Midwives have recommended IAP to prevent EOGBS since the early 1990s [11]. Following a large multicenter cohort study in 2002 [12] that suggested universal screening to be superior to risk-based screening, US guidance recommended universal screening (using a vaginal and a rectal swab) for GBS colonization at 35–37 weeks of gestation and among women with threatened preterm delivery and unknown colonization status, in addition to administration of high-dose intravenous benzylpenicillin or ampicillin in labor in those with a positive GBS swab [13, 14]. Additional risk factors for which IAP may be offered include women with GBS bacteriuria in the current pregnancy, a previous infant with GBS disease, vaginal or rectal swab positive for GBS in the current or a previous pregnancy, women with unknown colonization status, and intrapartum risk factors such as prolonged rupture of membranes or maternal intrapartum pyrexia above 38°C. In the United Kingdom, clinical risk factor screening (as outlined above, with the addition of preterm labor) is performed. Since the introduction of IAP policies [13, 14], culture-confirmed EOGBS disease in the United States declined from 1.7 per 1000 live births in the 1990s to 0.21 per 1000 live births in 2015 [7, 15, 16].

The use of clinical risk factor–based IAP strategies rather than universal screening is based on the belief that the introduction of routine microbiological screening may not reduce EOGBS-related mortality and morbidity sufficiently to be cost-effective. The recent National Screening Committee report assessing GBS screening expressed serious concern that it was not clear whether benefits associated with screening outweighed the harms for the majority of the population and that large numbers of women would be offered and take antibiotics when they do not need to, increasing potential risks associated with widespread antibiotic use in both women and infants [17]. A UK test accuracy study found that only 28.9% (89/307) of women with clinical risk factors actually carried GBS (whether tested by rapid intrapartum polymerase chain reaction [PCR]–based methods or following enriched culture medium of swabs in late pregnancy) and that 19% (205/1080) of women with no clinical risk factors carried GBS [18].

A recent Cochrane review that evaluated IAP for known maternal GBS colonization identified 4 randomized trials that involved 852 GBS-positive women (3 trials were more than 20 years old) and compared ampicillin or penicillin to no treatment. No clear differences in newborn deaths were found, although the occurrence of early GBS infection in the newborn was reduced with antibiotics (risk ratio [RR], 0.17; 95% CI, 0.04 to 0.74) [19]. The review was critical of the quality of the studies, including their small size, and considered there to be a high risk of bias in their methodology and execution, with further adequately sized, double-blind, controlled trials needed.

While there is broad agreement that IAP should be offered if risk factors are present, there is currently no formal international consensus as to whether IAP is best directed to women through universal screening or based on the presence of clinical risk factors. A European consortium has previously called for a universal intrapartum GBS screening strategy that uses rapid real-time PCR testing [20]. A review of universal antenatal culture-based screening for maternal GBS colonization to prevent EOGBS disease using program appraisal criteria for the UK National Screening Committee noted that the criteria for recommending a universal screening program were not currently met. The review concluded that further research would require randomized, controlled trial evidence, with economic modeling to evaluate the associated costs [21].

Several studies from the United States indicate that at least 40% of all infants are exposed to IAP for clinical reasons or because their mothers had a GBS-positive swab during pregnancy [22, 23]. Intrapartum antibiotics are delivered at a key time in the development of the infant’s intestinal microbiome. These microbiota may be important for metabolic, nutritional, physiological, and immunological development and rapidly evolve following birth. If the initial exposure to maternal microbiota is altered, such as following a caesarean birth, studies demonstrate differences in the type, variation, and distribution of organisms. The same may be true following IAP, and these effects could therefore have long-term health implications. The microbiome has been implicated in adult disease such as obesity, allergy and atopy, inflammatory bowel disease, and the development of colon cancer [24]. However, causal links have yet to be established, and there is a vital need to understand infant gut microbial community succession and to study the impact of early-life exposure to IAP on the processes involved in establishing a healthy microbiome.

Several recent studies indicate alterations in infant microbiota in IAP-exposed and IAP-unexposed infants up to 12 months of life. Two Canadian studies (Baby and Mi, CHILD) have recently published evidence that suggests early differences in the microbiota of low-risk term infants exposed to IAP compared to unexposed infants and those born by caesarean section [25, 26]. These studies suggest cumulative dysbiosis with IAP and caesarean section and modifying effects of breastfeeding. An Italian study of 84 infants followed for 30 days indicated reduced numbers of beneficial *Bifidobacterium* in IAP-exposed infants, which was further exacerbated in those who were fed formula rather than breast milk [27]. It is therefore possible that IAP may also alter the initial colonizing microbiota, and this could have long-term health implications. Several larger cohort studies are now underway to investigate the long-term effects of peripartum antibiotic exposure following caesarean section on the infant intestinal microbiome and future disease risk.

Box 1 summarizes research questions associated with GBS natural course and screening.

**Box 1: Research Questions Around Group B Streptococcus Natural Course and Screening**

• What is the clinical benefit and cost-effectiveness of universal screening (and treatment) for group B streptococcus (GBS) using the best-available microbiological tests to reduce early onset GBS-related sepsis, mortality, and morbidity compared to current risk factor–based screening?
• What are the medium- and long-term clinical sequelae and costs related to infants with early onset and late onset GBS disease, stratified by clinical presentation?
• How can existing datasets and/or routine data be used to collect this information?
• Does intrapartum antibiotic prophylaxis (IAP) have an impact on the infant’s microbiome and, if so, what is the clinical impact of this change on short- and long-term outcomes?
• What factors affect the adoption or uptake of different screening and testing approaches and of IAP after risk-based screening?

## TESTING STRATEGIES FOR BACTERIAL LOAD/VIRULENCE—COLONIZATION TO INVASIVE DISEASE

It is recognized that a major barrier to screening and prophylaxis for GBS disease is that current tests detect GBS colonization in pregnant women rather than predict infant invasive disease. A better understanding of the factors that determine bacterial virulence and host susceptibility is needed in order to develop a microbiological test that better targets the women (and babies) who require antibiotic prophylaxis and prevent the administration of unnecessary antibiotics to the women and babies who would otherwise remain well. This research should entail completion of biological studies before technological issues (eg, development of cheaper PCR-based tests that incorporate antibiotic sensitivities) are addressed and any new microbiological tests are clinically evaluated.

There is a paucity of data regarding bacterial factors that influence transmission of GBS from mother to fetus and neonate, and of those factors that maintain homeostasis in the infant intestine or cause invasive disease. Recently, a number of virulence factors and GBS lineages such as hypervirulence clonal complex 17 [28] and surface proteins (eg, Rib, Alp, and Pilus proteins) [29] have been implicated in increased disease risk and colonization persistence. Additionally, the initial inoculum (the woman’s bacterial load at the point of transmission) has also been associated with an increased risk of EOGBS [30]. Additional insights such as these could allow targeted implementation of IAP to only those women who carry the variants of GBS that are most likely to cause EOGBS, thus reducing the IAP currently offered to all women with GBS colonization. Even less is understood about LOGBS. It is unclear whether LOGBS is predominantly derived from environmental sources (horizontal transmission) or whether the infant gut harbors pathogens that cause episodes of LOGBS as a result of genetic alterations after transmission, as has been described with other pathogens such as pneumococcus [9, 31]. A recent study of LOGBS in a single neonatal unit using whole-genome sequencing (WGS) suggested that the majority of cases in that setting were likely to reflect nosocomial transmission [32]. Information about the characteristics of GBS that increase the risk of neonatal colonization and persistence may have important implications for more targeted IAP and for second-generation vaccine development. Such knowledge would also enable screening tests to be adapted toward these specific markers.

Development of tests that could reliably predict which women will transmit GBS to their child and which infants will go on to develop disease would be a substantial addition to current screening programs. Developing our understanding of the role of immunological factors and GBS strains on birth outcomes and combining this information with the population structure of GBS in colonization and disease will strengthen our knowledge of potential vaccine coverage and molecular diagnostic targets.

Several molecular diagnostic tools are emerging as potential candidates for more rapid identification of invasive GBS disease and more rapid identification of women who are GBS colonized. Rapid diagnostics for invasive disease include the MinIon, loop-mediated isothermal amplification [33], and optical immunoassays; those for rapid intrapartum colonization screening include PCR-based methods [34]. However, little data about their sensitivity and specificity for GBS detection in the clinical setting are publicly available. Recently, WGS was used to investigate GBS colonization factors, providing an opportunity to investigate beyond serotyping and overcoming some of the sensitivity issues that relate to the current latex agglutination tests [35]. WGS could be used to identify antimicrobial resistance genes that might be useful targets for any future screening in the context of penicillin allergy in order to make better choices around IAP. However, the methods remain cumbersome and cannot be developed in real time.

An alternative could be a PCR-based method that would target those genes identified by WGS as conferring clindamycin resistance, such as those developed for pneumococcal disease [36]. However, it is important to consider that looking at GBS genomics alone will not provide sufficient evidence for more targeted IAP. Host factors will also play an important role in identifying those who do and do not progress from colonization to disease.

Box 2 outlines the research questions surrounding GBS testing.

**Box 2: Research Questions Around Group B Streptococcus Testing**

• What factors in the mother, infant, and bacterium influence the development of invasive group B streptococcus (GBS) disease and how do these relate to the identification of maternal (or neonatal) colonization?
• Can a microbiological test that has sufficient accuracy and convenience (for women and the health service) be developed for clinical practice to detect GBS isolates that are likely to cause invasive disease rather than colonization alone and could the test be incorporated into the current healthcare system?
• Can rapid, sensitive diagnostics that improve the identification of infants with invasive GBS disease be developed?
• What is the appropriate reference standard against which to measure any new rapid diagnostic test?
• Could incorporation of antibiotic resistance genes into a polymerase chain reaction primer set enable development of a rapid test with additional information on antibiotic susceptibility in the context of penicillin allergy?

## GROUP B STREPTOCOCCUS VACCINES

Clinical evaluation of GBS vaccines using a reduction in invasive neonatal disease as a primary outcome requires large studies that are best carried out in settings with relatively high prevalence. It is estimated that an efficacy study of approximately 60 000 pregnant women in countries with a disease incidence of more than 1 in 1000 live births would be required to detect a 75% reduction in EOGBS and LOGBS disease [37]. This figure is based on the assumption that the vaccine would cover approximately 90% of circulating serotypes [37]. An alternative approach would be to establish immune correlates of protection based on vaccine or natural antibody studies such as the ones used for meningococcal and higher-valency formulations of pneumococcal polysaccharide-conjugate vaccines.

The issue for GBS, however, is that although there are data to support the concept of an immune correlate of protection [38], it is difficult to link this evidence to the vaccines currently in development. Several case-control studies have provided evidence that serocorrelates of protection against infant GBS disease are achievable [39–41]. These studies ranged in size from 25 000 to 140 000 pregnant women and captured between 33 and 109 cases of neonatal GBS disease. Each study provided evidence that higher antibody concentrations were linked to a reduced probability of contracting disease. However, no study was sufficiently powered to provide a definitive answer and, as the studies were all assessed using different assays and data analysis, it is not possible to compare and pool these results. There is an urgent need to develop a consensus around a validated serocorrelate of protection.

To facilitate more rapid licensure and availability of a GBS vaccine for prevention of early-onset and late-onset infant GBS disease, a serocorrelate of protection against neonatal invasive GBS disease will undoubtedly prove useful. However, when applying a serocorrelate of protection against a neonatal disease with a defined risk period where the prevention strategy is vaccinating the pregnant woman, additional factors including placental immunoglobulin G antibody transfer and antibody decay must be considered. These factors are important as a serocorrelate will likely need to demonstrate that vaccines can generate antibody titers in the mother that can be effectively transferred to and persist within the infant so that they are protected not only against early-onset but also late-onset disease.

It is not easy to determine a protective antibody concentration, as protective antibody concentrations may vary by serotype [40, 42] and the assessment of immunogenicity varies by the assay methods used [38, 42]. It is clear that a well-characterized assay that has been demonstrated to be robust, reproducible, specific, and precise is required.

Several initiatives are currently underway to facilitate the determination of a serocorrelate of protection against invasive disease, including standardization of assays [43]. Capsular polysaccharide CRM_197_ conjugate vaccine [44], tetanus toxoid protein conjugate vaccine, and an Alp/Rib protein adjuvanted vaccine are all in development [45].

Box 3 outlines research questions surrounding the development and testing of a GBS vaccine for use in the United Kingdom.

**Box 3: Development and Testing of A Vaccine For Group B Streptococcus**

• What are the serological correlates of protective immunity against invasive group B streptococcus (GBS) infection in UK women and infants?
• What are the safety, immunogenicity, and effects on colonization of a multivalent GBS vaccine given from 28 weeks of pregnancy to pregnant women and their infants, and what is the tolerability, acceptability, and safety profile of the vaccine in the UK population?
• What are the attitudes and knowledge of parents-to-be and healthcare professionals related to antenatal vaccination for GBS and how can we learn from recent implementation of other vaccines for pregnancy (eg, pertussis) to inform this?
• How do these attitudes and knowledge vary across groups of different ethnicity and socioeconomic status and how would we work with these groups to maximize engagement, including attendance and take-up?
• What is the role of midwives, family physicians, obstetricians, and patient support groups in ensuring good take-up?

## CONCLUSIONS

Many questions remain that require more evidence if we are to truly reduce the burden and impact of GBS disease in the United Kingdom and other countries with high disease burden. It should be a priority for the research community to come together to answer these complex questions.

## Notes

***Acknowledgments.***The following persons attended 1 or both workshops: Professor Lucy Chappell (chair), Dr Mark Turner (deputy chair), Dr Baharak Afshar, Professor Debra Bick, Professor Peter Brocklehurst, Dr Paul Cosford, Dr Kirsty Le-Doare, Dr Fiona Denison, Ms Elizabeth Duff, Professor Androulla Efstratiou, Professor Andrew Gorringe, Professor Paul Heath, Dr Rhona Hughes, Dr Theresa Lamagni, Dr Shamez Ladhani, Professor Anne Mackie, Ms Sarah Manson, Professor Neena Modi, Dr Natalie Owen, Dr Heather Payne, Mrs Jane Plumb, Professor Philip Steer, Professor Catherine Peckham, Dr Esther Robinson, Dr Farah Seedat, Dr Sian Taylor-Phillips, Dr Caroline Trotter, Professor Tom Walley, Professor Chris Whitty.

***Financial support.***K. L. D. is supported by a Bill and Melinda Gates Foundation award (OPP1153630).

***Potential conflicts of interest.***K. L. D. has received funds from Pfizer outside the submitted work to attend a meeting in April 2016. P. T. H. reports grants from Pfizer Inc and GlaxoSmithKline outside the submitted work; is a scientific advisory group member to the World Health Organization project Developing a Value Proposition for Vaccines Against Group B Streptococcal Disease; and is a member of the UK Royal College of Obstetrics and Gynaecology Prevention of Early-onset Neonatal Group B Streptococcal Disease Guideline. P. B. reports personal fees from Medical Research Council (MRC)/National Institute for Healthcare Research (NIHR); grants from MRC, NIHR Health Services and Delivery Research (HS&DR), NIHR Health Technology Assessment (HTA), and Wellcome Trust; and personal fees from Biotest AG outside the submitted work. All other authors report no potential conflicts. All authors have submitted the ICMJE Form for Disclosure of Potential Conflicts of Interest. Conflicts that the editors consider relevant to the content of the manuscript have been disclosed.
